# Association between dietary inflammatory index and all-cause mortality in US adults with dermatitis: a population-based cohort study

**DOI:** 10.3389/fnut.2024.1469630

**Published:** 2024-10-11

**Authors:** Yan Liu, Jie Liao, Jieyi Zhang, Rang Zhou, Weiqi Li, Yuanrong Tao, Yuesi Qin

**Affiliations:** ^1^Department of Clinical Nutrition, Chengdu First People's Hospital, Chengdu, China; ^2^West China School of Public Health and West China Fourth Hospital, Sichuan University, Chengdu, China; ^3^State Key Laboratory of Oral Diseases, National Clinical Research Center for Oral Diseases, West China Hospital of Stomatology, Sichuan University, Chengdu, China; ^4^Department of Dermatology, Chengdu Second People's Hospital, Chengdu, China; ^5^Department of Integrative Dermatology, Chengdu First People's Hospital, Chengdu, China

**Keywords:** dietary inflammatory index, dermatitis, all-cause mortality, cohort study, National Health and Nutrition Examination Survey (NHANES)

## Abstract

**Background:**

While dermatitis management is primarily symptomatic, the role of diet in symptom exacerbation and improvement is increasingly recognized. The dietary inflammatory index (DII), a quantitative assessment tool for dietary inflammatory potential, has been associated with various chronic diseases but remains understudied in dermatitis. This cohort study investigated the association between DII scores and all-cause mortality among patients with dermatitis.

**Methods:**

The study included 1,074 patients with dermatitis and complete dietary intake records from the National Health and Nutrition Examination Survey (NHANES) 1999–2004. The DII scores were calculated using two 24-h dietary recalls and dietary supplement intakes and the consumption of 28 foods with known pro- or anti-inflammatory properties. All-cause mortality information was from the National Death Index, censored on December 31, 2019. Multivariable Cox hazards regression models, restricted cubic spline (RCS) models, and subgroup analyses were employed to evaluate the association of DII with all-cause mortality, adjusting for potential confounders.

**Results:**

The 1,074 patients were divided into DII tertiles (T1: *n* = 358, median age 40 years, DII −3.91, 0.06; T2: *n* = 296, median age 40 years, DII 0.06, 1.88; T3: *n* = 237, median age 39 years, DII 1.88, 4.39). The study revealed a positive correlation between higher DII scores and increased all-cause mortality risk among patients with dermatitis (fully-adjusted model, HR = 1.13, 95% CI 1.02, 1.27, *p* = 0.026). This association was more pronounced in adults over 45 years, with the highest DII tertile indicating a 2.42-fold increased mortality risk (95% CI 1.15, 5.07, *p* = 0.019) compared with the lowest tertile. The RCS model confirmed a linear dose-response trend (*p* for non-linear = 0.183), validating the relationship.

**Conclusion:**

Elevated DII scores are associated with an increased risk of all-cause mortality in patients with dermatitis, suggesting that the dietary inflammatory potential may impact health outcomes in this population. The findings underscore the importance of dietary interventions in dermatitis management, especially for middle-aged and older adults. Future research with larger cohorts and a longer follow-up is warranted to validate the findings.

## 1 Introduction

Dermatitis refers to disorders characterized by an inflammation response in the skin, causing red rashes, dry skin, itchiness, and scaliness. The most prevalent types of dermatitis are atopic dermatitis (eczema), contact dermatitis, and seborrheic dermatitis. Dermatitis is a leading chronic inflammatory skin disease worldwide, affecting ~20% of children (1–5) and 3% of adults (1, 3, 4, 6–8). Dermatitis is non-life-threatening in nature but cannot be cured, leading to a lifelong struggle with symptom management, symptom recurrence, increased risk of inflammation-related comorbidities, and a poor quality of life (9–11).

The pathophysiology of dermatitis is complex, involving genetic and environmental factors that influence epithelial function and alter immune responses (12). Current treatments primarily address symptoms, such as reducing itching and skin dryness, rather than offering a cure (13–16).

Nutrition is a central lifestyle habit with profound potential impacts on human health (17), including in the management and prognosis of dermatitis (3, 18, 19). Gluten or wheat protein drive the helper T (Th)1/Th2/Th17 immune response, whereas dietary fiber and omega-3 fatty acids negatively regulate inflammatory cytokine production (20). Nevertheless, despite the known influence of diet on inflammation and dermatitis development (19, 21–23), data on dietary practices and their relation with dermatitis prognosis are scarce. It is well-established that certain components of the human diet, including additives in processed foods (24–27), gluten (28, 29), and deficiencies in nutrients like vitamin D (30–33), can trigger dermatitis and contribute to inflammation. Despite this knowledge, the overall impact of diet on the management of dermatitis remains underexplored. There is a notable gap in research concerning how the balance of pro- and anti-inflammatory elements in the human diet affects dermatitis prognosis.

The Dietary Inflammation Index (DII) was developed by Shivappa et al. in 2009 and updated in 2014. It provides a quantitative assessment of dietary inflammatory potential (34). A high DII indicates a pro-inflammatory diet, while a low DII suggests an anti-inflammatory diet (34). The DII is correlated with inflammatory biomarkers such as C-reactive protein (CRP), interleukin (IL)-6, and tumor necrosis factor (TNF)-α in various ethnic groups (35–39), and those inflammatory biomarkers contribute to the development and progression of dermatitis (40–42). Exacerbated systemic inflammation can impair skin barrier function and contribute to lesion progression (2, 12, 18, 19, 43–47). Many studies explored the association between DII and chronic diseases like metabolic syndrome, cardiovascular diseases, neurodegenerative diseases, and cancer (48–56), but the association between the DII and dermatitis prognosis remains unknown.

Therefore, this study aimed to investigate the association between the DII and all-cause mortality in patients with dermatitis. The study could provide data on whether dietary interventions could improve dermatitis prognosis.

## 2 Materials and methods

### 2.1 Data sources

The National Health and Nutrition Examination Survey (NHANES) is a cross-sectional study conducted biennially by the Centers for Disease Control and Prevention (CDC) to assess the health and nutritional status of the non-institutionalized U.S. population. The survey encompasses a comprehensive set of sociodemographic, dietary, and health-related data collected through interviews, physical examinations, and laboratory tests from representative samples across ~15 U.S. counties. The study protocol, including data extraction and usage for this project, was approved by the National Center for Health Statistics (NCHS) ethics review board. All participants provided written informed consent. Detailed information is available on the NHANES Informed Consent webpage: https://www.cdc.gov/nchs/nhanes/irba98.htm. This study used data from the National Health and Nutrition Examination Survey (NHANES), managed by the National Center for Health Statistics (NCHS). The NHANES is conducted under strict protocols to ensure the privacy and confidentiality of its participants. All data are anonymized, and participants are assured that their personal information will not be disclosed. The investigators had no access to any identifying information. The survey adheres to the privacy rules of the Health Insurance Portability and Accountability Act (HIPAA) and has been reviewed and approved by the National Center for Health Statistics Institutional Review Board. For more detailed information on NHANES data access and privacy policies, please visit the official NHANES website at: https://www.cdc.gov/nchs/nhanes/index.htm.

### 2.2 Study population

**Figure 1:**
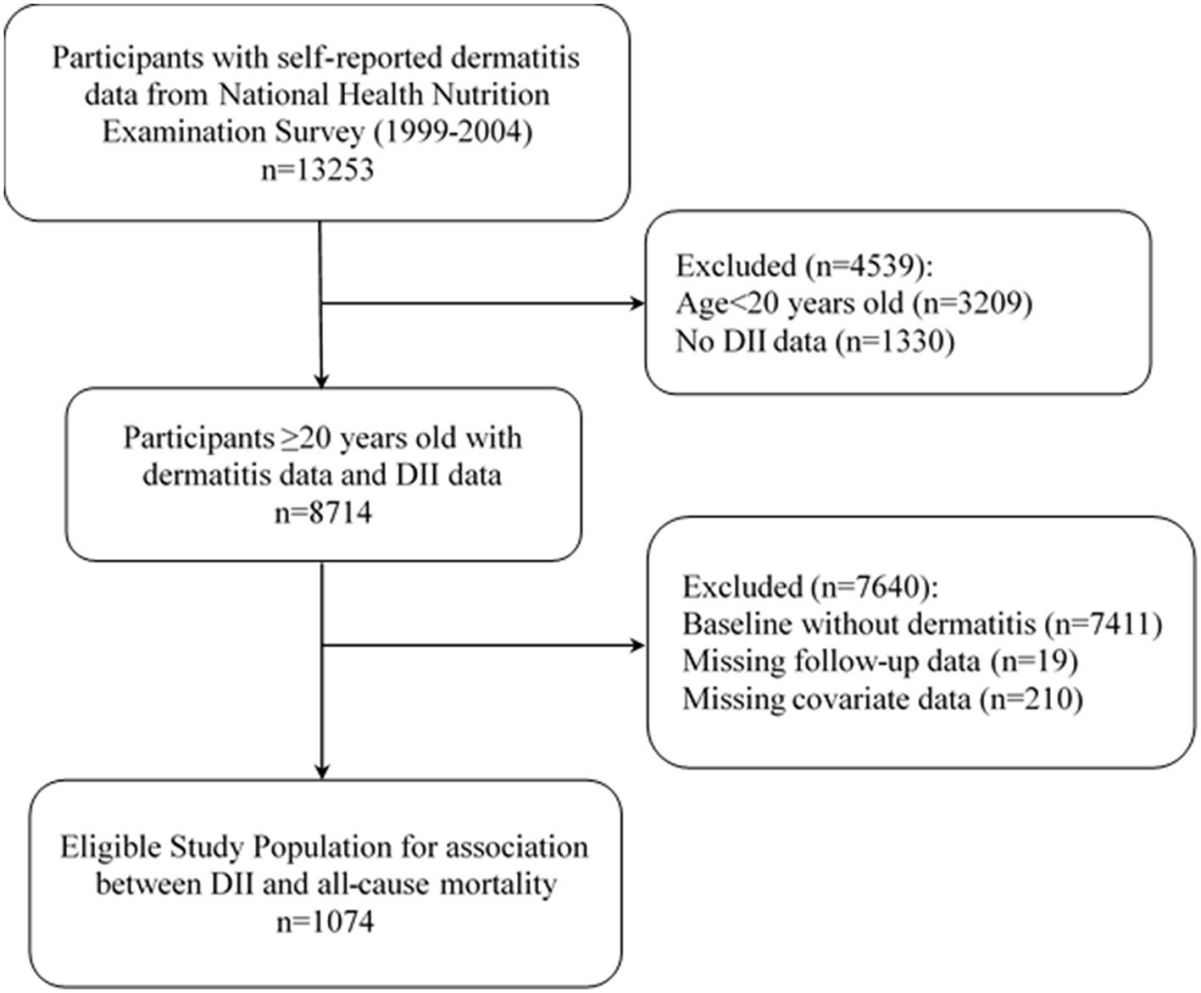
Flow chart of the screening process for the selection of eligible participants. DII, Dietary inflammatory index.

For the present study, data were extracted from 13,253 participants with complete dermatitis records from the NHANES database for 1999 to 2004, encompassing three 2-year cycles. The participants aged < 20 years (*n* = 3,209) or without two comprehensive records of 24-h dietary intake data for DII calculation (*n* = 1,330) were excluded. Then, 7,411 participants without dermatitis were excluded. Those with missing follow-up data (*n* = 19) or missing covariable information involved in this study (*n* = 210) were also excluded. Finally, 1,074 patients with dermatitis were included in this study to analyze the association between DII and all-cause mortality, all with a complete dataset for the purposes of the present study. Figure 1 illustrates the participant inclusion flowchart.

### 2.3 Measurements

#### 2.3.1 Definition of dermatitis

Dermatitis was assessed using the NHANES self-reported questionnaires. A positive answer to the question “During the past 12 months, that is, since DISPLAY CURRENT MONTH a year ago, have you/has SP had dermatitis, eczema, or any other type of red, inflamed skin rash?” was recognized as dermatitis.

#### 2.3.2 Assessment of diet and calculation of the DII

The daily total intake of nutrients was calculated by averaging two 24-h dietary data points and adding the average daily dietary supplement intake. The dietary information was collected via two 24-h diet recalls conducted by the Nutrition Methodology Working Group of the NHANES study (57). All the dietary data were validated by the Nutrition Methodology Working Group (58). The first diet recall was conducted in person in the mobile examination center, while the second recall was added by telephone interview ~3–10 days after the first recall. Using the Automated Multiple Pass Method (AMPM), all foods and beverages consumed during the previous day were recorded. A standard set of measuring guides was used to help the respondent report the volume and dimensions of the food items consumed. Food intakes were coded, and nutrient values were determined using the United States Department of Agriculture (USDA) Food and Nutrient Database for Dietary Studies (FNDDS), versions 1.0–5.0 (59). FNDDS provides the nutritional values of each food and beverage reported by NHANES. Nutrient intake was calculated based on the type and amount of food consumed. NHANES participants also reported on the dietary supplements they had taken in the past 30 days in an internal interview. For each nutrient, the daily dose was calculated by combining product information on the frequency of use, ingredients, quantities, and units per serving. The intake of each product was summarized to estimate the total daily dose of each nutrient for each participant. The total daily intake of nutrients was obtained by summing the average of two 24-h dietary data points and the average daily intake of dietary supplements (57).

The DII was calculated for each participant based on their dietary intake data according to the method developed by Shivappa et al. (34); it involves assigning pro-inflammatory or anti-inflammatory scores to various dietary components and then aggregating these scores to obtain a single DII value for each individual. Twenty-eight food parameters were used to calculate DII in the present study, including energy, carbohydrates, protein, total fat, alcohol, fiber, cholesterol, saturated fat, monounsaturated fatty acids, polyunsaturated fatty acids, omega-3 fatty acids, omega-6 fatty acids, niacin, vitamin A, vitamin D, vitamin E, thiamin (vitamin B1), riboflavin (vitamin B2), vitamin B6, vitamin B12, vitamin C, iron, magnesium, zinc, selenium, folic acid, beta-carotene, and caffeine. Previous studies have illustrated the stable predictive ability of DII when using 28 food parameters (35). The calculation of the DII is a meticulous process that unfolds in several steps. Initially, the nutrient intakes are standardized through z-transformation, which involves comparing each nutrient's intake to the mean and standard deviation as recorded in the global dietary standard library, encompassing 45 dietary nutrients. This normalization process adjusts the data to a mean of zero and a standard deviation of one. Subsequently, to centralize the data, the z-scores are multiplied by two and then adjusted by subtracting one, yielding a percentage result that reflects the deviation from the norm regarding inflammatory potential. Following this, the standardized nutrient values are each multiplied by their corresponding impact scores, which quantify the specific contribution of each nutrient to the overall inflammatory effect of the diet. Finally, the individual DII scores for all nutrients consumed are aggregated to determine a person's total DII score, reflecting the overall inflammatory potential of the diet, with higher scores indicating a more pro-inflammatory diet.

#### 2.3.3 All-cause mortality

Referring to previous studies by Li et al. (60) and Hicks et al. (61), the present study used the mortality data obtained from the Linked Mortality Files (LMFs) provided by the National Center for Health Statistics (NCHS). The NCHS has linked the NHANES III and continuous NHANES data from 1999 to 2018 to the National Death Index (NDI). Eligibility for mortality linkage was determined by the availability of sufficient identifying data among all survey participants. The underlying causes of death were ascertained from death certificates, encompassing both specified and unknown causes. Participants without any death indication were presumed alive and were censored as of December 31, 2019 (https://www.cdc.gov/nchs/data-linkage/mortality-public.htm). After linking the mortality data from LMFs, the present study could be considered a prospective cohort study (60, 61). The vital status codes from the LMFs were used to determine the mortality status in the statistical analyses. The dates of the initial inclusion in the study were defined as the participants' baseline. The person-years were determined from baseline to the date of death, date of loss to follow-up, or December 31, 2019, whichever occurred first.

#### 2.3.4 Covariables

The covariables considered in the analysis included age, sex, race/ethnicity, education level, health insurance, physical activity, hypertension (HTN) and hyperlipidemia (HPL), diabetes, and chronic kidney disease (CKD). Age was divided into the following categories: 20–29, 30–44, and 45–59 years old. Race/ethnicity was categorized into Non-Hispanic White, Non-Hispanic Black, Hispanic, and other races (62). Education level was divided into the following categories: those not graduated from high school, high school graduates or equally, and college graduates or above (27). Health insurance was divided into private (including any private health insurance or single-service plan), public only (including Medicare, Medicaid, military healthcare, or other government programs), and none (63). Physical activity was classified as inactive (without self-reported leisure time physical activity), recommended (leisure time moderate activity ≥5 times or vigorous activity ≥3 times per week), and insufficient (active but did not meet the recommended criteria) (64). The diagnostic criteria for HTN were an average systolic blood pressure of ≥140 mmHg or diastolic blood pressure ≥90 mmHg, self-reported doctor informed of HTN, or receiving blood pressure control measures such as taking antihypertensive medication. HPL was defined as serum cholesterol ≥200 mg/dL, doctor informed of hyperlipidemia, or receiving cholesterol control measures. Chronic kidney disease was defined based on glomerular filtration rate (GFR) < 60 mL/min/1.73 m^2^ or albuminuria >3 mg/mmol (65). More details of the methods used for covariables measurement can be found on the NHANES website.

### 2.4 Statistical analysis

Statistical analysis was conducted using weighted means ± standard error for continuous variables and frequencies and percentages for categorical variables. Differences across groups were assessed using independent *t*-tests for normally distributed continuous variables, Mann-Whitney *U*-tests for non-normally distributed continuous variables, and chi-squared tests for categorical variables. The relationship between the DII and the risk of all-cause mortality among patients with dermatitis was evaluated using a multivariable Cox regression model. Hazard ratios (HRs) with 95% confidence intervals (CIs) were calculated, considering DII as a continuous variable and as a categorical variable using tertiles, with the first tertile serving as the reference group. Four nested models were developed to adjust for an increasing number of covariables: Model 0 (unadjusted), Model 1 (adjusted for age, sex, and race), Model 2 (further adjusted for health insurance and physical activity), and Model 3 (fully adjusted for diabetes, HTN, HPL, and CKD).

Restricted cubic spline (RCS) models were used to explore the potential non-linear associations between DII and the risk of all-cause mortality. The robustness of the findings was tested through stratification by sex and age and by examining interactions between these factors and the DII-mortality relationship. All analyses were conducted using R version 4.2.2, with statistical significance defined as *p* < 0.05. Additional details on statistical methods and software packages used are available upon request.

## 3 Results

### 3.1 Basic characteristics of participants by DII tertiles

The study included 1,074 participants with a median age of 40 years, distributed nearly equally between males (43.4%) and females (56.6%). The range of the DII scores spanned from −3.91 to 4.39 (first tertile: −3.91 to 0.06; second tertile: 0.06 to 1.88; third tertile: 1.88 to 4.39). The distribution of the baseline characteristics across the three DII tertiles is shown in Table 1. Notably, the third tertile exhibited a higher prevalence of female participants, Non-Hispanic Black individuals, inactive physical status, lack of health insurance, and a greater incidence of HTN and CKD compared with the first tertile.

**Table 1:** DII tertile baseline characteristics distribution of the dermatitis patients aged 20 years and older from NHANES 1999–2004 [*n* (%) or M (P25, P75)].

**Characteristics**	**Dietary inflammatory index**				***p*-value**
	**All (*****n*** = **1,074)**	**T1 (*****n*** = **358)**	**T2 (*****n*** = **296)**	**T3 (*****n*** = **237)**	
		**(-3.91–0.06)**	**(0.06–1.88)**	**(1.88–4.39)**	
Age [median (IQR)]	40.00 [30.00, 48.00]	40.00 [31.00, 48.00]	40.00 [30.00, 49.00]	39.00 [29.00, 48.00]	0.576
Sex (%)					**< 0.001**
Male	466 (43.4)	204 (57.0)	167 (46.6)	95 (26.5)	
Female	608 (56.6)	154 (43.0)	191 (53.4)	263 (73.5)	
Race ethnicity (%)					0.214
Non-Hispanic White	655 (61.0)	229 (64.0)	215 (60.1)	211 (58.9)	
Non-Hispanic Black	173 (16.1)	42 (11.7)	62 (17.3)	69 (19.3)	
Hispanic	207 (19.3)	72 (20.1)	69 (19.3)	66 (18.4)	
Other race	39 (3.6)	15 (4.2)	12 (3.4)	12 (3.4)	
Physical status (%)					0.439
Inactive	125 (11.6)	36 (10.1)	39 (10.9)	50 (14.0)	
Insufficient	717 (66.8)	239 (66.8)	240 (67.0)	238 (66.5)	
Recommended	232 (21.6)	83 (23.2)	79 (22.1)	70 (19.6)	
Health insurance (%)					0.136
No insurance	215 (20.0)	61 (17.0)	81 (22.6)	73 (20.4)	
Public insurance	105 (9.8)	34 (9.5)	28 (7.8)	43 (12.0)	
Private insurance	754 (70.2)	263 (73.5)	249 (69.6)	242 (67.6)	
Hypertension (%)					0.193
No	766 (71.3)	259 (72.3)	264 (73.7)	243 (67.9)	
Yes	308 (28.7)	99 (27.7)	94 (26.3)	115 (32.1)	
Hyperlipidemia (%)					0.109
No	483 (45.0)	167 (46.6)	145 (40.5)	171 (47.8)	
Yes	591 (55.0)	191 (53.4)	213 (59.5)	187 (52.2)	
T2D (%)					0.352
No	992 (92.4)	325 (90.8)	332 (92.7)	335 (93.6)	
Yes	82 (7.6)	33 (9.2)	26 (7.3)	23 (6.4)	
CKD (%)					0.274
No	982 (91.4)	333 (93.0)	328 (91.6)	321 (89.7)	
Yes	92 (8.6)	25 (7.0)	30 (8.4)	37 (10.3)	

DII, Dietary Inflammatory Index; NHANES, National Health and Nutrition Examination Survey; IQR, Interquartile Range; T2D, Type 2 diabetes mellitus; CKD, Chronic Kidney Disease.

Education level: those not graduated from high school, high school graduates or equally, and college graduates or above. Health insurance: private (including any private health insurance or single-service plan), public only (including Medicare, Medicaid, military healthcare, or other government programs), and none. Physical activity: inactive (without self-reported leisure time physical activity), recommended (leisure time moderate activity ≥5 times or vigorous activity >3 times per week), and insufficient (active but did not meet the recommended criteria). The bold values signify statistical significance at *p* < 0.05.

### 3.2 Associations between DII and mortality

#### 3.2.1 Multivariable regression analysis

As shown in Table 2, when considering the DII scores as a continuous variable in the initial unadjusted Cox regression model (Model 0), no significant associations were observed between the DII scores and the risk of all-cause mortality. After adjusting for age, sex, and race in Model 1, a significant positive correlation was identified, which was consistent and remained statistically significant in Models 2 and 3 (HR = 1.13, 95% CI 1.01, 1.27 for Model 2, and HR = 1.13, 95% CI 1.02, 1.27 for Model 3), meaning that each increased unit of DII increases the mortality risk by 1.13 times. When analyzed as a categorical variable by tertiles, the third tertile of DII scores showed a non-significant trend toward an elevated risk of all-cause mortality compared with the first tertile.

**Table 2:** Association between the DII scores and all-cause mortality among patients with dermatitis, NHANES 1999–2004.

	**Model 0**	**Model 1**	**Model 2**	**Model 3**
	**HR (95% CI)** ***p-*****value**	**HR (95% CI)** ***p-*****value**	**HR (95% CI)** ***p-*****value**	**HR (95% CI)** ***p-*****value**
Continuous	1.07 (0.96, 1.19) 0.221	1.13 (1.01, 1.27) **0.037**	1.13 (1.01, 1.27) **0.031**	1.13 (1.02, 1.27) **0.026**
**DII tertile**				
1st tertile	ref = 1.00	ref = 1.00	ref = 1.00	ref = 1.00
2nd tertile	1.07 (0.66, 1.75) 0.780	1.11 (0.67, 1.81) 0.692	1.17 (0.71, 1.92) 0.539	1.33 (0.80, 2.20) 0.271
3rd tertile	1.25 (0.77, 2.01) 0.364	1.59 (0.97, 2.62) 0.066	1.58 (0.96, 2.60) 0.072	1.59 (0.96, 2.63) 0.700
*P* for trend	0.368	0.071	0.085	0.085

DII, Dietary Inflammatory Index; NHANES, National Health and Nutrition Examination Survey; HR, hazard ratio; CI, confidence interval; ref, reference.

Model 0: Unadjusted model.

Model 1: Adjusted for age, sex, and race.

Model 2: Adjusted for age, sex, race, health insurance, and physical activity.

Model 3: Adjusted for age, sex, race, health insurance, physical activity, diabetes, hypertension, hyperlipidemia, and chronic kidney disease. The bold values signify statistical significance at *p* < 0.05.

#### 3.2.2 Subgroup analysis

The DII levels and their correlations with the mortality risk among patients with dermatitis were analyzed by stratifying for sex, age, HTN, HPL, and T2D (Table 3). The results of the subgroup analyses indicated a significant association between DII scores and all-cause mortality risk among patients with dermatitis over the age of 45, with HTN or without T2D. A refined analysis within the 45–59 age group (Table 4) showed a significant positive association between DII as a continuous variable and all-cause mortality across all four models. Notably, in this age group, the second and third tertiles presented a significantly heightened risk of all-cause mortality after a comprehensive adjustment for confounding factors. The HRs with corresponding 95% CIs were 2.21 (1.06, 4.62) for the second tertile and 2.42 (1.15, 5.07) for the third tertile, respectively.

**Table 3:** Stratified analysis of the DII and all-cause mortality among patients with dermatitis, NHANES 1999–2004.

**Variables**	**Dietary inflammatory index**			***P* for trend**
	**1st tertile**	**2nd tertile**	**3rd tertile**	
	**HR (95% CI)**	**HR (95% CI)**	**HR (95% CI)**	
**Sex (%)**				
Male	Ref = 1.00	1.055 (0.577, 1.929)	1.356 (0.728, 2.526)	0.357
Female	Ref = 1.00	2.618 (0.899, 7.627)	2.220 (0.799, 6.169)	0.189
**Age (%)**				
[20, 30]	Ref = 1.00	0.313 (0.028, 3.509)	0.249 (0.032, 1.917)	0.166
[30, 45]	Ref = 1.00	0.743 (0.316, 1.745)	1.010 (0.437, 2.332)	0.957
[45, 60]	Ref = 1.00	2.548 (1.219, 5.325)	2.666 (1.251, 5.680)	**0.013** ^*^
**Hypertension (%)**				
No	Ref = 1.00	1.142 (0.587, 2.224)	1.132 (0.562, 2.283)	0.714
Yes	Ref = 1.00	1.503 (0.689, 3.280)	2.301 (1.102, 4.803)	**0.025** ^*^
**Hyperlipidemia (%)**				
No	Ref = 1.00	2.022 (0.883, 4.633)	2.052 (0.887, 4.633)	0.096
Yes	Ref = 1.00	0.983 (0.508, 1.901)	1.526 (0.800, 2.912)	0.210
**T2D (%)**				
No	Ref = 1.00	1.609 (0.893, 2.900)	1.982 (1.090, 3.605)	**0.025** ^*^
Yes	Ref = 1.00	0.799 (0.219, 2.917)	1.103 (0.312, 3.896)	0.960

DII, Dietary Inflammatory Index; NHANES, National Health and Nutrition Examination Survey; HR, hazard ratio; CI, confidence interval; ref, reference; T2D, Type 2 diabetes mellitus.

Adjusted for age, sex, race, health insurance, physical activity, diabetes, hypertension, hyperlipidemia, and chronic kidney disease, the calibration factors are excepted from the stratification factor. The bold values signify statistical significance at *p* < 0.05. The symbol ^*^ indicates statistical significance at *p* < 0.05.

**Table 4:** Association between the DII scores and all-cause mortality among patients with dermatitis (age ≥ 45 years old), NHANES 1999–2004.

	**Model 0**	**Model 1**	**Model 2**	**Model 3**
	**HR (95% CI)** ***p-*****value**	**HR (95% CI)** ***p-*****value**	**HR (95% CI)** ***p-*****value**	**HR (95% CI)** ***p-*****value**
Continuous	1.19 (1.02, 1.38) 0.026	1.23 (1.05, 1.45) 0.011	1.25 (1.06, 1.47) 0.008	1.25 (1.06, 1.47) 0.007
**DII tertile**				
1st tertile	Ref = 1.00	Ref = 1.00	Ref = 1.00	Ref = 1.00
2nd tertile	1.86 (0.92, 3.75) 0.085	1.84 (0.91, 3.75) 0.091	1.87 (0.91, 3.82) 0.087	2.21 (1.06, 4.62) 0.034
3rd tertile	2.00 (1.00, 4.02) 0.052	2.29 (1.12, 4.70) 0.024	2.33 (1.13, 4.79) 0.022	2.42 (1.15, 5.07) 0.019
*P* for trend	0.057	0.024	0.023	0.022

DII: Dietary Inflammatory Index; NHANES, National Health and Nutrition Examination Survey; HR, hazard ratio; CI, confidence interval; ref, reference.

Model 0: Unadjusted model.

Model 1: Adjusted for age, sex, and race.

Model 2: Adjusted for age, sex, race, health insurance, and physical activity.

Model 3: Adjusted for age, sex, race, health insurance, physical activity, diabetes, hypertension, hyperlipidemia, and chronic kidney disease.

#### 3.2.3 Non-linear association research between DII and mortality

**Figure 2:**
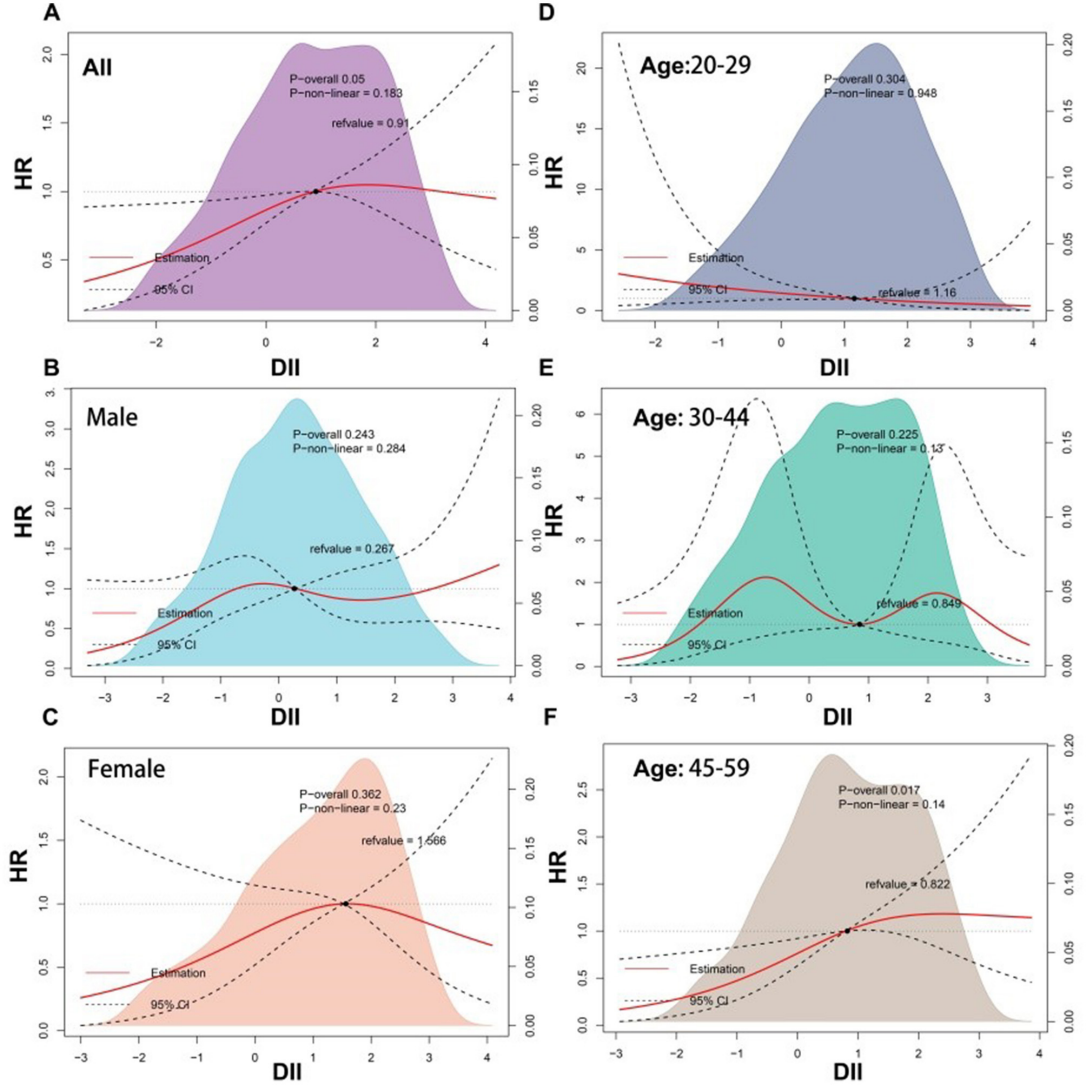
Restricted cubic spline (RCS) analyses between DII and all-cause mortality of participants with dermatitis. DII, dietary inflammatory index.

The RCS results are shown in Figure 2, suggesting a positive linear correlation between DII scores and the risk of all-cause mortality among patients with dermatitis (*P* for non-linear = 0.183). However, this correlation varied by sex, with males showing an increasing trend in mortality risk as DII scores exceeded 1 (*P* for non-linearity = 0.284) and females showing the opposite trend (*P* for non-linear = 0.23), although neither reached statistical significance.

## 4 Discussion

In this study, using data from a nationally representative cohort, the correlation between the DII scores and the risk of all-cause mortality was investigated in patients with dermatitis. The findings strongly suggest a significant association between higher DII scores and an elevated risk of all-cause mortality among patients with dermatitis. It was accentuated by a positive linear dose-response trend observed through the RCS model. Notably, this correlation exhibits age-specific variability, with a pronounced positive association in adults over 45 years of age, contrasting with the lack of such a trend in younger patients. No previous studies examined the association between the DII scores and the mortality of patients with dermatitis.

Diet and nutrition as potential triggers of atopic dermatitis have long been debated in the literature, as well as the attempt to attribute diet to a therapeutic effect on atopic dermatitis. The intricate relationship between food antigens and the worsening of dermatitis symptoms, particularly in the context of IgE-mediated food allergies such as gluten, peanuts, and cow's milk, has been highlighted in numerous studies (66–68). Expanding on the allergenic effects, a growing body of evidence suggests that specific dietary patterns and nutrients influence the onset and progression of dermatitis. The International Study of Asthma and Allergies in Childhood (ISAAC) presented compelling evidence that regular consumption of fresh fruit offers a significant protective effect against dermatitis. At the same time, frequent fast food intake correlates with increased disease severity (69). This observation is further supported by the inverse relationship between the prevalence of atopic dermatitis and the intake of vegetables, cereal proteins, and fish, both fresh and frozen, as reported in another ISAAC study (70, 71). The protective effects of these foods are hypothesized to be due to their rich content of n-3 polyunsaturated fatty acids (PUFA), vitamin C, or phytochemicals, all of which are known for their anti-inflammatory properties (72–75). The significance of n-3 fatty acids in dermatitis mitigation is further underscored by intervention studies, such as a 12-week double-blind trial demonstrating the therapeutic impact of fish oil supplementation on the clinical parameters of dermatitis, including scaling, pruritus and overall severity (76). In addition, vitamin D, an established anti-inflammatory nutrient, has been identified in a meta-analysis by Kim et al. (77) as being negatively associated with the prevalence of atopic dermatitis. Patients with lower serum vitamin D levels exhibited improved SCORAD and EASI scores following supplementation, indicating the nutrient's potential role in disease severity amelioration. On the other hand, the higher incidence of dermatitis in developed countries, as opposed to developing ones, may reflect the impact of dietary habits. A Western diet, replete with processed foods, red and processed meats, and refined grains, is suggested to contribute to the increased prevalence and progression of dermatitis due to its pro-inflammatory nature (53, 78–80). This dietary pattern elevates the risk of chronic inflammation and associated diseases. Therefore, it is reasonable to believe that the overall pro-inflammatory or anti-inflammatory capacity of the diet may influence the prognosis of dermatitis.

As a robust assessment tool, DII has been extensively studied and validated to evaluate the potential impact of the overall diet on the inflammation levels in the human body (52, 53). A substantial body of research consistently revealed a significant positive relationship between the DII and all-cause mortality across diverse populations (53, 81–83). A comprehensive study involving 14,460 patients with HPL in the United States of America (USA) has established a significant positive linear correlation between the DII scores and the risk of all-cause mortality (84). After a median follow-up period of 211 months, it was observed that individuals with elevated DII faced an increased risk of death from all causes. This finding is consistent with previous research that demonstrated a similar association in different cohorts, including populations with cardiovascular disease (54, 85), T2D (63, 86, 87), hyperuricemia (88), and older adults with HTN (48). In addition, a positive relationship between DII scores and all-cause mortality has also been observed among patients with asthma (56), suggesting that inflammatory diets could exacerbate asthma symptoms and affect survival rates. The findings from the present study concord with the studies mentioned above, suggesting that an anti-inflammatory diet may reduce the all-cause mortality associated with various chronic diseases.

The potential mechanisms by which a high DII diet increases the all-cause mortality rate in dermatitis patients are likely linked to the exacerbation of systemic chronic inflammation. A pro-inflammatory diet, characterized by a higher DII, is associated with increased levels of inflammatory markers such as TNF-α, CRP, and IL-6. These inflammatory mediators are known to play a pivotal role in the pathogenesis of dermatitis (40–42). Exacerbated systemic inflammation from a pro-inflammatory diet can impair skin barrier function, enhance susceptibility to infections, and contribute to the progression of skin lesions, thereby potentially leading to a worsened disease course (2, 12, 18, 19, 43–47) and increased mortality risk in these patients. Furthermore, the sustained inflammatory state may also affect other organ systems, increasing the risk of comorbidities (89–93), accelerating biological aging (94), and overall mortality (52, 53, 95). Schutte et al. (96) showed that a high DII score (i.e., a pro-inflammatory diet) was associated with worse atopic outcomes in children. They reduced the systemic buffer against dermatitis environmental triggers.

The subgroup analysis indicated a significant and stable positive association between DII and all-cause mortality among patients with dermatitis aged 45–60 years but not among those younger than 45 years. It could be attributed to a heightened sensitivity to pro-inflammatory diets in older individuals compared with younger people. On the other hand, it is plausible that older people have been exposed to pro-inflammatory diets for extended periods, leading to a more substantial cumulative effect on systemic inflammation. Furthermore, this age group may also contend with additional unfavorable health factors, such as increased family responsibilities and work-related stress, which can exacerbate inflammation and its adverse effects on health. The prevalence rates of chronic conditions like cardiovascular diseases, HTN, HPL, and T2D, known to be increased by inflammatory processes, are also higher in older groups (97, 98). Higher prevalence rates of those inflammation-related comorbidities could be related to the increase in mortality observed in older patients. A pro-inflammatory diet could potentially exacerbate the progression of these chronic diseases, thereby elevating the risk of all-cause mortality. Another explanation for the observed age-specific association could be the small number of mortality events in the 20–44 age group. It results in insufficient statistical power to detect a significant link between DII and all-cause mortality. It is also important to consider that the impact of inflammation on health may be more pronounced with age, suggesting that the physiological response to inflammation, including the activation of immune pathways and the subsequent tissue damage, may be more detrimental in older individuals. In summary, while these potential explanations provide a foundation for understanding the observed age-specific associations, further research is needed to clarify how DII influences mortality in dermatitis patients of different age groups. Understanding these nuances can inform tailored dietary recommendations and interventions to mitigate the impact of inflammation on health outcomes in dermatitis patients across various ages. The subgroup analysis indicated a significant association between DII and all-cause mortality among dermatitis patients with HTN, one of the reasons for this result might be that, compared with the general population, HTN patients have higher levels of serum inflammatory markers, including CRP, high-sensitivity CRP, fibrinogen, and IL-6, which are also associated with target organ damage and the risk of future cardiovascular events (99). As introduced earlier, DII is positively correlated with the abovementioned inflammatory markers.

An HR of 1.13, as observed in the whole study population, indicates that for each 1 increased unit of DII, the risk of mortality is increased by 1.13 times. Therefore, it could be recommended that patients with dermatitis reduce their consumption of pro-inflammatory nutrients and increase their intake of anti-inflammatory nutrients to decrease their DII. Therefore, for patients with dermatitis in the USA, particularly those in the middle or advanced age groups, dietary guidelines should prioritize the reduction of the DII to optimize patient prognosis. This reduction could be pursued by selecting a diet replete with antioxidants, dietary fiber, and unsaturated fatty acids, as found abundantly in vegetables, fruits, nuts, whole grains, deep-sea fish, and flaxseeds. In parallel, it would be crucial to diminish the consumption of processed foods typically laden with sugars, sodium, and fats, as well as calorific carbohydrates, which are high in empty calories. Although the DII is calculated based on specific pro- and anti-inflammatory nutrients, it does not consider the general pattern of the diet. Indeed, high sugar intake, rather than high-calorie intake, exacerbates skin inflammation in mouse models (100). In addition, a long-term Western diet activates Th2 and Th17 cells, which are associated with specific dermatitis subtypes (101). Hence, although the DII can be helpful in guiding dietary choices in terms of nutrients, additional studies are necessary to investigate the general dietary patterns on the prognosis of dermatitis.

This study possessed several methodological strengths. The data in this analysis were derived from the NHANES, a database renowned for its extensive sample size, thereby enhancing the findings generalizable for the USA population. In addition, the study incorporated adjustments for many potential confounding factors within the sensitivity analysis model, aimed at substantiating the robustness of the results and mitigating the likelihood of spurious causal inferences. Nonetheless, the study was not devoid of limitations. Firstly, the DII scores were calculated based on two 24-h dietary recalls, inevitably introducing recall bias. Secondly, given this is a cross-sectional observational study, definitive causality could not be established, and there is a risk of reverse causality. Thirdly, despite the correction for many pertinent confounding factors, the potential for residual confounding persisted, such as energy intake. The study included all factors collected in the NHANES survey that may be related to diet. Of course, several potentially relevant covariates to dermatitis prognosis that were not included in the NHANES design could not be included. Fourthly, in NHANES, the data on dermatitis is self-reported, and there are no data on the types of dermatitis, preventing analyses on the influence of the type of dermatitis on patient prognosis. Lastly, the study evaluated the initial DII score in correlation with prognosis. Yet, the dynamic surveillance of DII scores throughout the follow-up period is critical for a comprehensive assessment. Future research employing larger cohorts, extended observation periods, and randomized controlled trials was warranted to validate and expand upon these findings. Longitudinal data would be necessary to determine the nature of the relationship between the DII and mortality in patients with dermatitis. Future studies should also include more precise dietary data to allow the determination of general dietary patterns and a more precise DII calculation.

## 5 Conclusion

In the USA, an elevated DII score was found to be correlated with an increased risk of all-cause mortality among patients with dermatitis aged 20–60 years. This correlation exhibits age-specific variability, with a pronounced positive association in adults 45–60. Therefore, for dermatitis sufferers within the USA, particularly those in the middle to advanced age group, dietary guidelines should prioritize the reduction of the DII.

## Data Availability

The datasets presented in this study can be found in online repositories. The names of the repository/repositories and accession number(s) can be found in the article/Supplementary material.
