# Supplementary material for: Association between dietary inflammatory index and all-cause mortality in US adults with dermatitis: a population-based cohort study
Source: Front Nutr. 2024 Oct 11;11:1469630. doi: 10.3389/fnut.2024.1469630 (PMC11502388; doi:10.3389/fnut.2024.1469630)
Supplement: Supplementary file 1 [file Data_Sheet_1.docx]

Supplementary Material

**Supplementary Table 1.** Baseline characteristics according to all-cause mortality, NHANES 1999-2004.

| **Variables** | **Alive**  **(N=973)** | **Death**  **(N=101)** | ***p* value** |
| --- | --- | --- | --- |
| DII (median [IQR]) | 0.86 [-0.50, 2.31] | 1.12 [-0.30, 2.40] | 0.349 |
| Age (median [IQR]) | 39.00 [29.00, 48.00] | 46.00 [42.00, 54.00] | <0.001 |
| Sex (%) |  |  | <0.001 |
| Male | 401 (41.2) | 65 (64.4) |  |
| Female | 572 (58.8) | 36 (35.6) |  |
| Race ethnicity (%) |  |  | 0.260 |
| Non-Hispanic White | 596 (61.3) | 59 (58.4) |  |
| Non-Hispanic Black | 150 (15.4) | 23 (22.8) |  |
| Hispanic | 191 (19.6) | 16 (15.8) |  |
| Other races | 36 (3.7) | 3 (3.0) |  |
| Physical status (%) |  |  | 0.119 |
| Inactive | 107 (11.0) | 18 (17.8) |  |
| Insufficient | 653 (67.1) | 64 (63.4) |  |
| Recommended | 213 (21.9) | 19 (18.8) |  |
| Health insurance (%) |  |  | <0.001 |
| No insurance | 198 (20.3) | 17 (16.8) |  |
| Public insurance | 78 ( 8.0) | 27 (26.7) |  |
| Private insurance | 697 (71.6) | 57 (56.4) |  |
| Hypertension (%) |  |  | <0.001 |
| No | 715 (73.5) | 51 (50.5) |  |
| Yes | 258 (26.5) | 50 (49.5) |  |
| Hyperlipidemia (%) |  |  | 0.686 |
| No | 440 (45.2) | 43 (42.6) |  |
| Yes | 533 (54.8) | 58 (57.4) |  |
| T2D (%) |  |  | <0.001 |
| No | 912 (93.7) | 80 (79.2) |  |
| Yes | 61 ( 6.3) | 21 (20.8) |  |
| CKD (%) |  |  | <0.001 |
| No | 901 (92.6) | 81 (80.2) |  |
| Yes | 72 ( 7.4) | 20 (19.8) |  |

**Supplementary Table 2.** Association between the DII scores and all-cause mortality among patients with dermatitis (age ≥ 20 & age < 30 years old), NHANES 1999-2004.

|  | **Model 0**  **HR (95% CI) *p* value** | **Model 1**  **HR (95% CI) *p* value** | **Model 2**  **HR (95% CI) *p* value** | **Model 3**  **HR (95% CI) *p* value** |
| --- | --- | --- | --- | --- |
| Continuous | 0.76 (0.52, 1.13) 0.172 | 0.79 (0.52, 1.21) 0.282 | 0.78 (0.52, 1.17) 0.230 | 0.73 (0.48, 1.09) 0.125 |
| DII tertile |  |  |  |  |
| 1st tertile | ref = 1.00 | ref = 1.00 | ref = 1.00 | ref = 1.00 |
| 2nd tertile | 1.86 (0.92, 3.75) 0.085 | 0.28 (0.03, 2.56) 0.260 | 0.22 (0.02, 2.39) 0.214 | 0.27 (0.02, 2.99) 0.283 |
| 3rd tertile | 2.00 (1.00, 4.02) 0.052 | 0.73 (0.12, 4.43) 0.735 | 0.58 (0.08, 4.20) 0.587 | 0.36 (0.04, 3.47) 0.376 |
| *P* for trend | 0.396 | 0.596 | 0.509 | 0.299 |

**Supplementary Table 3.** Association between the DII scores and all-cause mortality among patients with dermatitis (age ≥ 30 & age < 45 years old), NHANES 1999-2004.

|  | **Model 0**  **HR (95% CI) *p* value** | **Model 1**  **HR (95% CI) *p* value** | **Model 2**  **HR (95% CI) *p* value** | **Model 3**  **HR (95% CI) *p* value** |
| --- | --- | --- | --- | --- |
| Continuous | 1.01 (0.85, 1.21) 0.874 | 1.09 (0.90, 1.32) 0.374 | 1.05 (0.88, 1.25) 0.598 | 1.04 (0.86, 1.26) 0.684 |
| DII tertile |  |  |  |  |
| 1st tertile | ref = 1.00 | ref = 1.00 | ref = 1.00 | ref = 1.00 |
| 2nd tertile | 0.61 (0.27, 1.39) 0.237 | 0.60 (0.26, 1.39) 0.233 | 0.61 (0.26, 1.42) 0.250 | 0.71 (0.30, 1.68) 0.442 |
| 3rd tertile | 0.91 (0.43, 1.91) 0.795 | 1.29 (0.59, 2.83) 0.530 | 1.02 (0.47, 2.24) 0.955 | 1.03 (0.45, 2.36) 0.938 |
| *P* for trend | 0.770 | 0.664 | 0.953 | 0.986 |


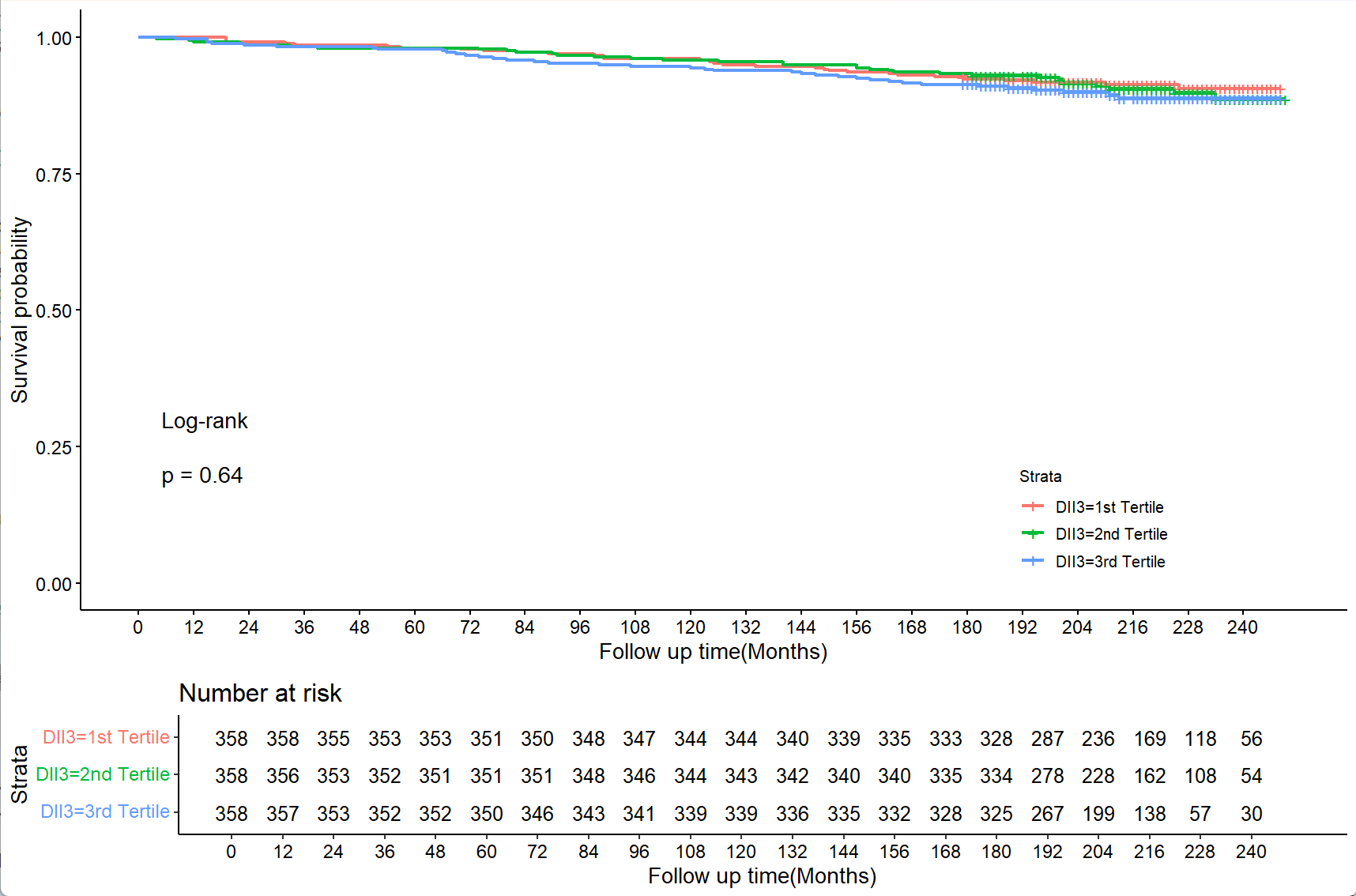


**Supplementary Figure 1.** Kaplan-Meier survival curves for all-cause mortality based on the Dietary Inflammatory Index among dermatitis patients
